# Probable Congenital Babesiosis in Infant, New Jersey, USA

**DOI:** 10.3201/eid1505.070808

**Published:** 2009-05

**Authors:** Sonia Sethi, David Alcid, Hemant Kesarwala, Robert W. Tolan

**Affiliations:** The Children’s Hospital at Monmouth Medical Center, Long Branch, New Jersey, USA (S. Sethi); Saint Peter’s University Hospital, New Brunswick, New Jersey, USA (D. Alcid, H. Kesarwala, R.W. Tolan, Jr.); Robert Wood Johnson Medical School, New Brunswick (D. Alcid); Drexel University College of Medicine, Philadelphia, Pennsylvania, USA (H. Kesarwala, R.W. Tolan, Jr.)

**Keywords:** Vector-borne infections, parasites, Babesia microti, babesiosis, congenital, hemolytic anemia, infant, newborn, New Jersey, dispatch

## Abstract

Only 2 neonates with transplacentally or perinatally acquired (congenital) babesiosis have been reported. We describe a probable third congenital case of babesiosis in a 26-day-old infant; transmission was determined on the basis of a blood smear from the infant (15% parasitemia) and serologic results from the infant and mother.

Victor Babes first described the pathogen of babesiosis in 1888 (*1*). Babesiosis is a tick-borne malaria-like illness transmitted by the same *Ixodes* spp. ticks that transmit *Borrelia burgdorferi* (*2*). It is endemic to the northeastern and northwestern United States and also occurs in Europe and parts of Asia. Babesiosis is an intraerythrocytic parasitic infection that ranges from subclinical to severe (possibly fatal) disease with fever, thrombocytopenia, hemolytic anemia, and hyperbilirubinemia. Appropriate antimicrobial drug therapy, transfusion, and exchange transfusion remain the mainstays of treatment.

Babesiosis occurs rarely among neonates, although it is gaining increasing attention as an emerging tick-borne zoonosis. In 1987, Esernio-Jenssen et al. (*3*) reported an apparent case of transplacentally or perinatally transmitted congenital babesiosis. In 1997, New et al. (*4*) reported another case. We describe a third case of probable congenital babesiosis in a 26-day-old infant with 15% parasitemia. She was treated successfully with atovaquone (Mepron; GlaxoSmithKline, Research Triangle Park, NC, USA) and azithromycin (Zithromax; Pfizer, New York, NY, USA).

## The Case

A 26-day-old, 8-pound, full-term infant girl was transferred to Saint Peter’s University Hospital for evaluation of fever and hyperbilirubinemia. For 1 week, she was not feeding well and was gagging and irritable. On the day of admission, her mother noted fever and yellow eyes. The mother (a migrant crop worker) reported having had an uneventful pregnancy, labor, and delivery, except for having been bitten by 2 ticks at 8 months’ gestation while picking crops in New Jersey. She did not seek treatment. The mother had not traveled elsewhere in the United States during her pregnancy. Knowledge about earlier travel to *Babesia*-endemic areas would have been helpful in understanding the mother’s infection, but this information was unavailable. The infant had no history of tick exposure; she had been outdoors only for visits to the pediatrician.

Physical examination showed an alert but pale infant weighing 4.4 kg; her temperature was 101.8°F (38.7°C), pulse rate 160/min, respiratory rate 36/min, blood pressure 90/40 mm Hg, and oxygen saturation 99% while breathing room air. Her conjunctivae were icteric. Her liver and spleen were palpable 4 cm and 5 cm below their respective costal margins. No hemorrhagic lesions or tick bites were noted. The rest of her physical examination findings were unremarkable except for a diaper rash.

Initial laboratory findings included a hemoglobin level of 8.8 g/dL (indices within normal limits); leukocyte count of 9.0/mm^3^ with 3% bands, 18% neutrophils, 72% lymphocytes, 7% monocytes; and platelet count of 34,000/mm^3^. Blood chemistry concentrations included total and indirect bilirubin 5.9 mg/dL (reference range 0.1–1.2 mg/dL); alanine aminotransferase 18 IU/L; aspartate aminotransferase 53 IU/L; alkaline phosphatase 108 IU/L; blood urea nitrogen 6 mg/dL; creatinine 0.3 mg/dL; and C-reactive protein 54 mg/dL (reference range 1.0–10.0 mg/dL). Peripheral blood smear demonstrated evidence of hemolysis and was consistent with *Babesia microti* infection (although *B. duncani* is indistinguishable from *B. microti* on peripheral smear) and ≈15% parasitemia (Figure).

**Figure:**
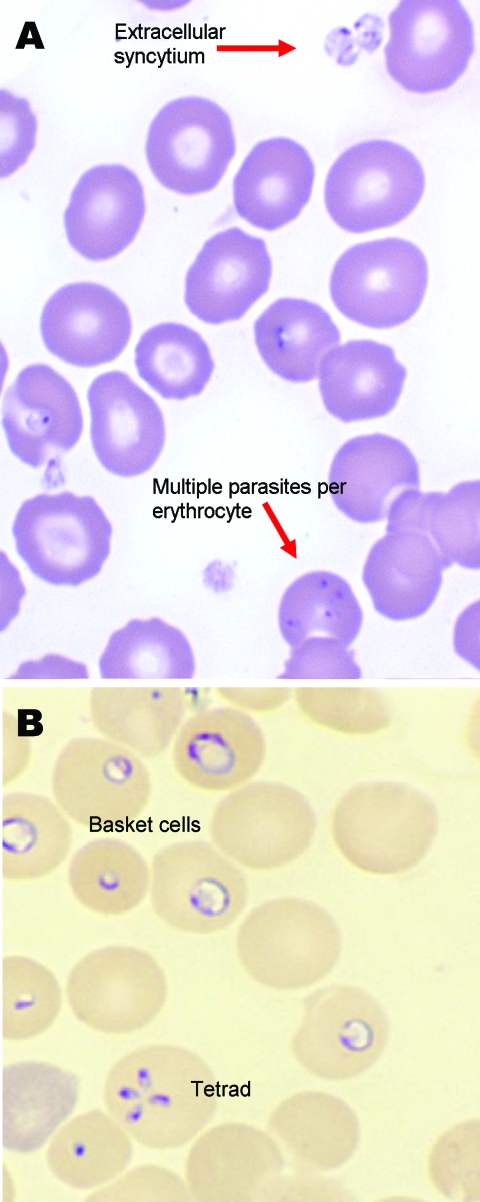
Giemsa-stained (A) and Wright-stained (B) peripheral blood smear from a newborn with probable *Babesia microti* infection. Parasitemia was estimated in this newborn at ≈15% based on the number of parasites per 200 leukocytes counted. The smear demonstrated thrombocytopenia and parasites of variable size and morphologic appearance and an absence of pigment. Magnification ×1,000.

Subsequently, the infant’s lactate dehydrogenase concentration was found to be 1,912 IU/L (reference range 313–618 IU/L) and later rose to 2,535 IU/L (Table 1). The infant’s *Babesia* immunoglobulin (Ig) G and IgM titers by immunofluorescent antibody (IFA), which are genus specific but not species specific, were 256 (reference <16) and 40 (reference <20), respectively (both tests were performed by Quest Diagnostics–Nichols Institute, Chantilly, VA, USA). Lyme IgG Western blot plus 2 Lyme IgM Western blots, performed early during hospitalization and just before discharge, were negative. The mother’s peripheral blood smear did not show any parasites, but her *Babesia* IgG and IgM titers by IFA were >1,024 and 80, respectively, and her Lyme serology was positive. The mother refused additional testing. Despite the variability in sensitivity and specificity of commercially available serologic tests (particularly the IFA for *Babesia* IgM), *Babesia* serologic results were not confirmed at a reference laboratory. Species-specific PCR was not performed.

**Table 1:** Clinical and laboratory data for infant with probable congenital babesiosis*

Clinical/laboratory data	Day of hospitalization							
	1	2	3	4	5	6	7	8
Maximum daily temperature, ºF	102.7	101.9	100.6	98.0	98.8	99.6	99.4	98.8
Hepatomegaly, cm below costal margin	4	4	4	5	Stable	Stable	Smaller	Smaller
Splenomegaly, cm below costal margin	5	5	5	5	Stable	Stable	Smaller	Smaller
Lowest daily hemoglobin level, g/dL	8.8	8.1	7.0†	9.8	8.6	7.4	8.5	9.6
Lowest daily platelet count, 10^3^/mm^3^	34	30	23	34	45	112	185	340
Parasitemia, %	15.2	Present‡	Present‡	2	<1	NA	0	NA
Highest daily total bilirubin level, mg/dL	5.9§	NA	3.6	3.9	2.7	2.1	1.7	1.3
Highest daily lactate dehydrogenase level, IU/L	NA	NA	1,912¶	2,481	2,535	2,286	2,239	1,566
Highest daily C-reactive protein level, mg/dL	54#	NA	130	82	62	39	NA	NA
Treatment	CRO, ATO, AZI	CRO, ATO, AZI	CRO, ATO, AZI, PRBC	CRO, ATO, AZI	CRO, ATO, AZI	CRO, ATO, AZI	CRO, ATO, AZI	CRO, ATO, AZI

*NA, not assessed; CRO, ceftriaxone sodium; ATO, atovaquone; AZI, azithromycin; PRBC, packed red blood cells. †Before PRBC transfusion. ‡Not quantified. §Reference range 0.1–1.2 mg/dL. ¶Reference range 313–618 IU/L. #Reference range 1.0–10.0 mg/dL.

 After concluding that this infant had probable congenital babesiosis, we began treating her with oral atovaquone (40 mg/kg/d) in 2 divided doses and azithromycin (12 mg/kg/d) once per day. The infant received 1 transfusion with packed red blood cells on hospital day 3 because of continued hemolysis, but she did not require exchange transfusion despite having a high initial parasite count. The infant’s parasitemia decreased rapidly, and she responded well to treatment (Table 1). She was discharged after 8 days and was to complete a 10-day course of atovaquone and azithromycin (which were well tolerated); she was subsequently lost to follow-up.

## Conclusions

Of 10 cases of babesiosis in neonates that have been reviewed (*5*), 2 were congenital (*3**,**4*), 2 were transmitted by a tick bite (*6*), and 6 were associated with transfusions (*5**,**7**–**9*). The 2 congenital cases (*3*,*4*) are compared to our probable congenital case (Table 2). All 10 of the affected neonates were reported to have <9% parasitemia (*5*). The illness ranged from no symptoms in 2 infants transfused with contaminated blood (*8*) to symptomatic disease (as in our infant) with fever and hepatosplenomegaly in 5 of 7 (71%), hemolytic anemia in 8 of 10 (80%), indirect hyperbilirubinemia in 4 of 5 (80%), and thrombocytopenia in 7 of 9 (78%) (*5*). Five of 8 (63%) patients required erythrocyte transfusion (*5*). The infant we describe had all of these manifestations as well as a higher parasite count than described previously (*5*). Clearly, the spectrum of neonatal babesiosis is variable and must be more fully elucidated, as must determinants of the illness’s clinical course and parasite clearance. In neonates, the degree of parasitemia may not parallel the severity of the babesiosis.

**Table 2:** Selected clinical data from the first 2 reported cases of congenital babesiosis (*3*,*4*) and the probable case described in this article*

Clinical data	Reference case 1	Reference case 2	Present case
Infant’s age at time of illness	30 d	5 wk	26 d
Time of maternal tick bite before delivery	1 wk	7 wk	4 wk
Serologic test results for *Babesia* spp.			
Mother	Pos	Pos	Pos
Infant	Pos	Pos	Pos
Clinical findings	Fever, irritability, pallor, hepatosplenomegaly	Lethargy, poor feeding, pallor	Fever, poor feeding, irritability, pallor, scleral icterus, hepatosplenomegaly
Parasitemia, %	5	4.4	15
Treatment (duration)	Ampicillin and gentamicin (3 d); clindamycin and quinine sulfate (10 d)	Clindamycin and quinine sulfate (12 d); azithromycin (10 d)	Ceftriaxone (8 d); atovaquone and azithromycin (10 d)

*All infants’ history of tick exposure was negative, and all recovered.

The combination of quinine sulfate and clindamycin hydrochloride for treatment of a newborn with transfusion-associated babesiosis was described in 1982 and subsequently became the first accepted treatment (*7*). A combination of azithromycin with atovaquone for 7 to 10 days has emerged as an alternative regimen (*8**,**10**–**11*), having been used successfully in 2 neonates (*8**,**10*) and several adults (*11*) in whom it appears to be safe and effective. Finally, the addition of azithromycin or atovaquone to the clindamycin hydrochloride plus quinine sulfate regimen has been proposed (*2**,**8*), particularly if parasitemia is slow to resolve.

Recently, our understanding of babesiosis and the methods of testing for it have improved dramatically. Because babesiosis (and congenital babesiosis) is an emerging tick-borne zoonosis, it is worthwhile to review the state-of-the-art approach to its diagnosis in the context of the limitations to diagnosis inherent in this particular case, including its retrospective nature, the mother’s lack of insurance and resultant unwillingness to undergo any additional laboratory testing, and the loss to follow-up of the infant and her migrant family.

Diagnosis of congenital babesiosis requires definitive evidence of babesiosis, including evidence from reference laboratory species-specific IFA testing, PCR confirmation, and evidence from reference laboratory evaluation of peripheral blood smears, particularly blood smears with high parasitemia (necessary because of the numerous species of *Babesia* endemic to the United States, including *B. microti*, *B. divergens*–like, *B. duncani*, MO-1, CA-1, and WA-1). Accurate diagnosis also requires collection of extensive epidemiologic information about patients with suspected infections, including their recent and remote travel history, exposure to ticks, transfusion or transplant. Follow-up for recrudescence is important, particularly for the immunocompromised patient. Our report of a probable third case of congenital babesiosis illustrates the variability in the manifestations and clinical course of the illness, suggesting a need for improvement in how the disease is recognized and for evaluation of current treatment modalities.
